# Endometriosis: From Genes to Global Burden

**DOI:** 10.3390/ijms27010151

**Published:** 2025-12-23

**Authors:** Pawel Kordowitzki, Liam P. Kelley, Sylvia Mechsner

**Affiliations:** 1 Department of Basic and Preclinical Sciences, Nicolaus Copernicus University, 87-100 Torun, Poland; 2 Department of Gynaecology, European Competence Centre for Ovarian Cancer, Charité Medical University, Augustenburgerplatz 1, 13353 Berlin, Germany; sylvia.mechsner@charite.de; 3 Program in Bioinformatics, Faculty of Computing and Data Sciences, Boston University, Boston, MA 02215, USA; kelley@bu.edu

**Keywords:** endometriosis, psychosomatic, pain, anxiety, global disease burden, Disability-Adjusted Life Years, years lived with disability

## Abstract

Endometriosis has a significant impact on the social, psychological, psychosomatic, and physical aspects of women’s lives. There is increasing evidence that endometriosis has to be seen as a systemic and complex disorder with a multifactorial etiology, accompanied by numerous other pathologies, such as mental disorders and even cancer. Herein, we analyzed Disability-Adjusted Life Years (DALYs) and Years Lived with Disability (YLDs) generated from the Global Burden of Disease Study (GBD 2021), which are key metrics used to measure the worldwide impact of diseases. Besides, differential gene expression data generated from the Turku Endomet Database were calculated. Briefly, log2-transformed gene expression counts were investigated using linear modeling with the function expression ~ condition to generate log2 fold changes and *p*-values for each gene. This enabled a precise comparative analysis of mRNA expression levels between control endometrium and various endometriosis-affected tissues, including ovarian endometrioma, peritoneal lesions, and deep endometriosis. Expression patterns of specific genes related to pain and malignant turnover within endometriosis samples and controls have been analyzed. The identification of upregulated genes like *FOS*, *DES*, *SIRT1*, *SBDS*, *SRF*, *SPN*, *P2RX1*, *TEAD3*, and *SLITRK3*, alongside downregulated genes such as *KIF22*, *KIF25*, *GAS2L2*, and *HINT3*, highlights a broad transcriptional reprogramming within endometriotic tissues. The clustering analysis, which reveals pain-related genes (*SRP14*/*BMF*, *GDAP1*, *MLLT10*, *BSN*, and *NGF*), further solidifies the genetic basis for the chronic and often debilitating pain experienced by patients with endometriosis. In 2021, women with endometriosis experienced the highest rates of total YLDs at 19.98%, with anxiety contributing 17.21% and major depression 8.12%, equating to mean YLDs of 15–24 years. In conclusion, our findings reinforce the need for adopting a holistic, psychosomatic approach to managing endometriosis. The identified genetic markers related to pain provide a biological basis for the profound physical suffering. At the same time, the robust DALYs and YLDs data quantify the devastating impact on mental health, particularly highlighting the significant burden of depression and anxiety.

## 1. Introduction

**Figure 1:**
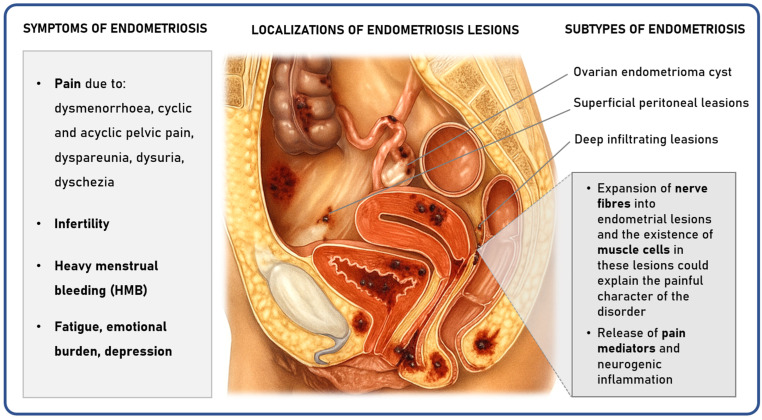
Scheme showing the common symptoms, localizations, and subtypes of endometriosis. The expansion of neural fibers into endometrial lesions of the peritoneum, along with the presence of muscle cells, could explain the painful character of the disorder and also have psychosomatic effects. Pain could be due to dysmenorrhea, cyclic and acyclic pelvic pain, dyspareunia, dysuria, and dyschezia.

In the management of endometriosis, understanding the patient’s perspective is crucial, as the disease has a profound impact on all aspects of life, including physical, psychological, and social. Endometriosis is defined by the presence of endometrial-like tissue outside the uterine cavity, affecting approximately 1 in 10 women of reproductive age [1,2]. The disease can manifest in various locations (Figure 1), including the ovaries, the peritoneum, the pouch of Douglas, and the vesico-uterine space. Researchers postulate that the origin of endometriosis is due to retrograde menstruation, which leads to endometriotic lesions, followed by coelomic metaplasia, resulting in peritoneal lesions. Additionally, vascular and lymphatic metastasis can lead to extra-pelvic lesions [1]. Patients who have endometriosis face a plethora of stigma and mental health issues [3,4,5]. Stigma is an under-studied yet significant dimension of the endometriosis experience, and addressing it can improve patient well-being and clinical care [4]. Psychosomatic aspects of the disease highlight the profound connection between mental distress and the physical symptoms of endometriosis, suggesting that psychological factors can amplify or even trigger somatic complaints [4,6]. Anxiety and depression, commonly reported by individuals with endometriosis, are thought to influence inflammatory responses and pain sensitivity [3,7]. This complex connection of the aforementioned factors implies that the ongoing psychological strain experienced by endometriosis patients may intensify the severity and persistence of their physical symptoms, such as pain [8]. Conversely, the physical realities of endometriosis, particularly chronic pain and infertility, impose considerable psychological stress, creating a detrimental cycle where bodily and emotional health continuously reinforce each other [9,10]. The debilitating pain associated with endometriosis can prevent individuals from pursuing daily activities and may affect sexual health, potentially leading to mental health problems such as depression, frustration, and loneliness. Moreover, due to the societal normalization of women’s pain and stigma around menstrual issues, there is a lack of disease awareness among women, healthcare providers, and the public [11,12].

As it is common for endometriosis, there is an estrogen-driven chronic inflammatory state, which significantly impacts a woman’s physical and mental well-being, leading to dyspareunia, dysmenorrhea, pelvic pain, and infertility [13]. The widespread effects on daily activities, including work, social interactions, and relationships, exacerbate mental health challenges, often resulting in depression, anxiety, and a reduced quality of life [14]. Furthermore, this systemic inflammation contributes to comorbidities like migraines and autoimmune diseases, complicating the condition and necessitating a multidisciplinary therapeutic approach [15]. Although it is still a subject of ongoing research and debate, one cannot forget that endometriosis is also a potential risk factor for ovarian cancer [1,11,16,17,18]. Importantly, it needs to be emphasized that the absolute risk for women to develop ovarian cancer is 1.3%, and in patients with endometriosis, it increases to 1.8% [16]. In a recent study in which 450,906 patients with and without endometriosis were analyzed, a history of endometriosis conferred a 4.2-fold increased risk for ovarian cancer [19]. Moreover, the aforementioned study revealed that women with severe endometriosis face an approximately tenfold increased risk of ovarian cancer compared to their counterparts without endometriosis [19]. However, another study postulates that a significant number of patients with endometriosis never develop ovarian cancer. After the exclusion of concurrent diagnosis of endometriosis and ovarian cancer, the HR is 1.71 [20]. In this regard, contemporary research seeks to elucidate the genetic predispositions for endometriosis. Studies indicate a heritability of endometriosis of approximately 50%, although the exact genetic loci and their penetrance are still subjects of ongoing research [21]. This outstanding interplay of genetic, hormonal, and immunological factors contributes to the variable clinical presentation of endometriosis, often leading to severe pain and psychosomatic effects. The pervasive nature of endometriosis extends beyond its direct physiological manifestations, profoundly influencing the psychological well-being and overall quality of life for millions of women globally [22]. In this regard, Disability-Adjusted Life Years (DALYs) and Years Lived with Disability (YLDs) are key metrics used to measure the global burden of diseases, including endometriosis. DALYs are a comprehensive measure of overall disease burden, combining the impact of premature mortality and the effect of living with a disability [23]. DALYs represent the sum of two components, namely the ‘Years of Life Lost’, which quantifies the years of life lost due to premature death caused by a disease, and the YLDs- this component measures the years of healthy life lost due to living with a disease or its sequelae, weighted by the severity and duration of the disability [23].

We hypothesize that distinct gene expression signatures in endometriosis-affected tissues are associated with both the biological mechanisms underlying chronic pain and the pronounced mental health-related disability observed at the global population level, thereby linking molecular alterations to the extensive social, psychological, and physical burden of the disease. Therefore, by using publicly available databases [24,25,26], this article aims to synthesize the current understanding of the psychosomatic impacts of endometriosis, elucidating how genetic components, DALYs, and YLDS associated with the condition contribute to significant pain and psychological distress, including elevated rates of anxiety, depression, and diminished quality of life among affected women.

## 2. Results

### 2.1. Gene Expression in Patients Suffering from Endometriosis

**Figure 2:**
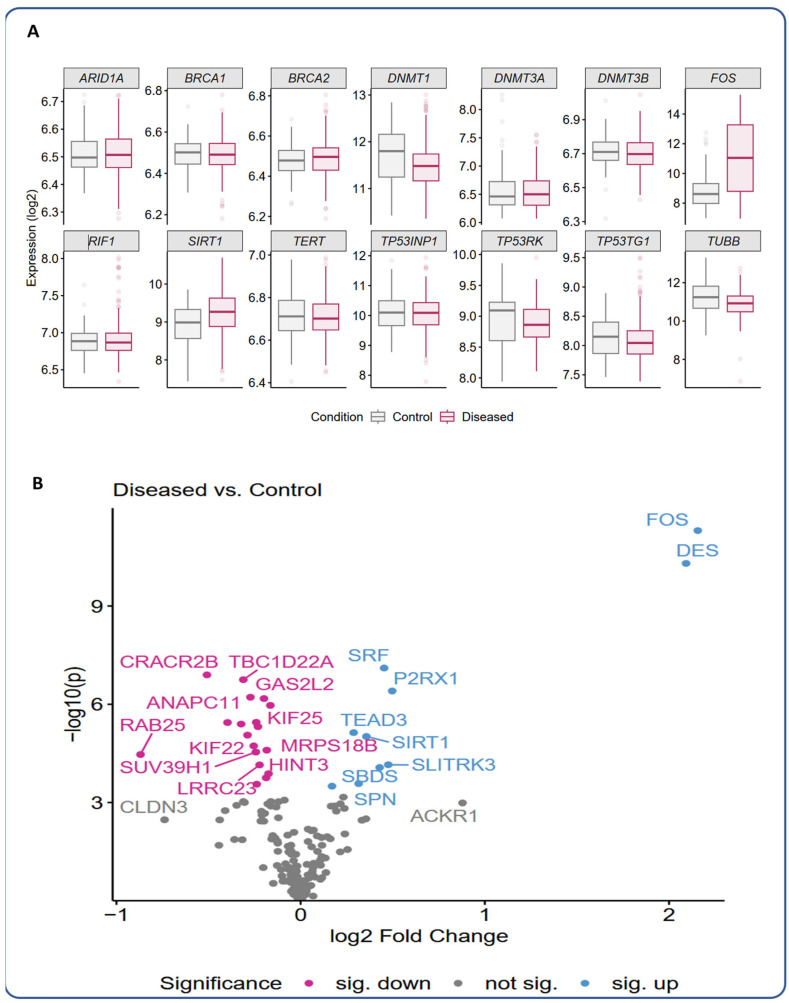
Gene expression analysis of selected genes in endometriosis and unaffected tissue based on the EndometDB. Panel (**A**) shows the expression of selected genes of interest in a box-plot diagram. In contrast, Panel (**B**) illustrates the significance of down- or upregulated genes in a volcano plot.

As a first step, the expression of selected genes of interest has been performed in endometriosis patients and respective unaffected controls, which are either related to pain, malignancy transformation, or are known markers for several ovarian cancer types and endometriosis-associated ovarian cancer (Figure 2). Here, the genes *FOS* and *DES* were particularly highly upregulated in endometriosis patients compared to controls (Figure 2B). The genes *SIRT1*, *SBDS*, *SRF*, *SPN*, *P2RX1*, *TEAD3*, and *SLITRK3* were also significantly upregulated. In contrast, some genes were significantly downregulated in endometriosis patients, among others *KIF22*, *KIF25*, *GAS2L2*, and *HINT3* (Figure 2B).

**Figure 3:**
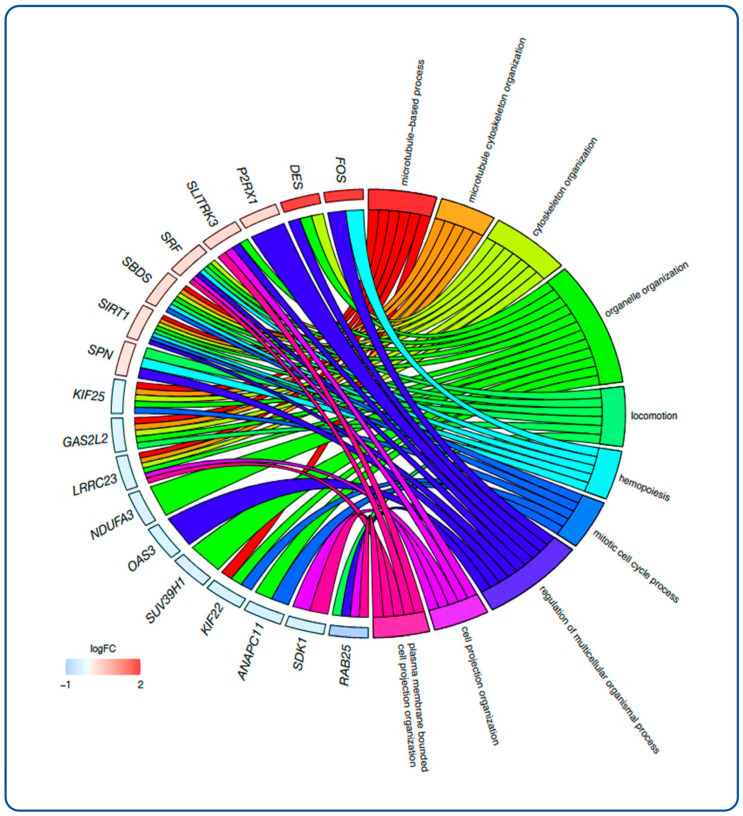
Chord diagram showing relationships between gene ontology (GO) terms and differently expressed genes (DEGs) in endometriosis and unaffected tissue based on the EndometDB. Enrichment analysis was performed on the set of DEGs found in endometriosis tissue relative to the control. Select GO terms in all exposures (FDR corrected *p* < 0.05) are shown. Genes are listed with log2 fold changes, listed from outer to inner circle; legend colors only specify fold change.

**Figure 4:**
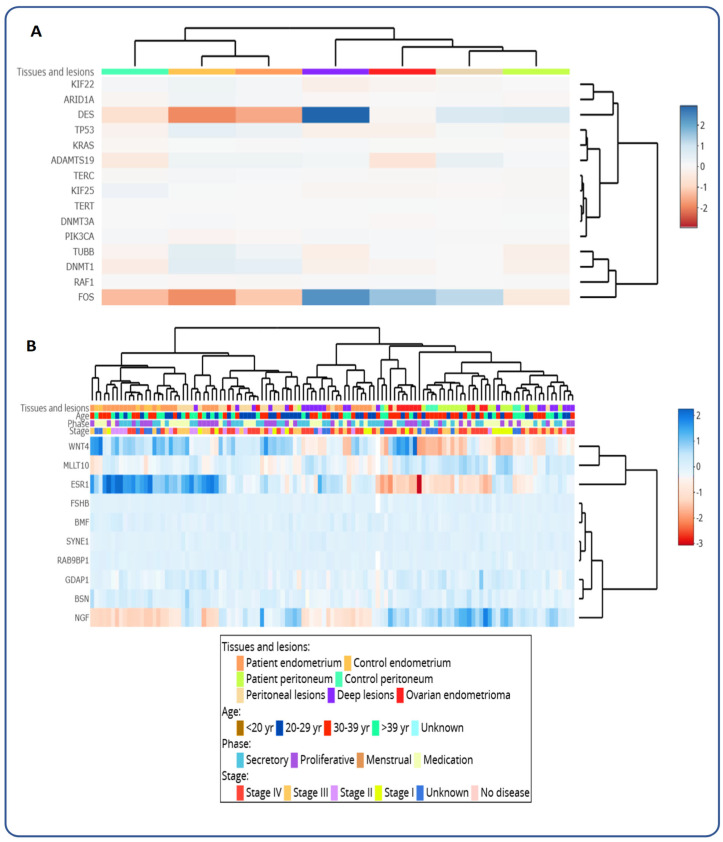
Unsupervised hierarchical clustering analysis of selected genes in endometriosis and unaffected tissue based on the EndometDB. (**A**) Unsupervised hierarchical clustering analysis of mRNA expression of selected differentially expressed genes with regards to tissues and lesions. (**B**) Unsupervised hierarchical clustering analysis of mRNA expression of selected differentially expressed genes with regards to tissues and lesions, patient age, cycle stage/phase, and endometriosis stage. The different clinical features of the samples (lesion/tissue type, age of patients with pre-selected grouping, hormonal stage, and Endometriosis stage) are presented in the heatmap. The Canberra distance metric, combined with Ward’s clustering method, was applied, revealing clusters corresponding to lesions and tissue types. The dendrogram on the *x*-axis shows the hierarchical relationship between the tissues and lesions, as well as the cycle phase and disease stage. The dendrogram on the *y*-axis shows the measure of similarities in the activation levels of the respective signaling pathway genes. The colors represent different tissues and lesions from both healthy controls and patients with endometriosis.

As shown in the chord diagram (Figure 3), which illustrates the relationships between Gene Ontology (GO) terms and differentially expressed genes (DEGs), the aforementioned gene *FOS* is highly associated with the regulation of multicellular organismal processes and hematopoiesis. The gene *DES* is linked to locomotion, cytoskeleton organization, and organelle organization. The genes *SLITRK3*, *SRF*, and *LRRC23* are associated with the organization of plasma membrane-bound cell projections and the arrangement of cell projections. In the unsupervised hierarchical clustering analysis of selected genes based on EndometDB (Figure 4), genes related to pain, such as *SRP14/BMF*, *GDAP1*, *MLLT10*, *BSN*, and *NGF*, have been investigated. The dendrogram on the *y*-axis shows the measure of similarities in the activation levels of the respective signaling (Figure 4B). Moreover, the gene *KRAS*, known to be mutated in endometriosis-associated ovarian cancer (EAOC), shows clustering with the gene *ADAMTS19*, which is known to promote proliferation and invasion in EAOC cells (Figure 4A).

### 2.2. Disability Adjusted Life Years and Years Lived with Disability

**Figure 5:**
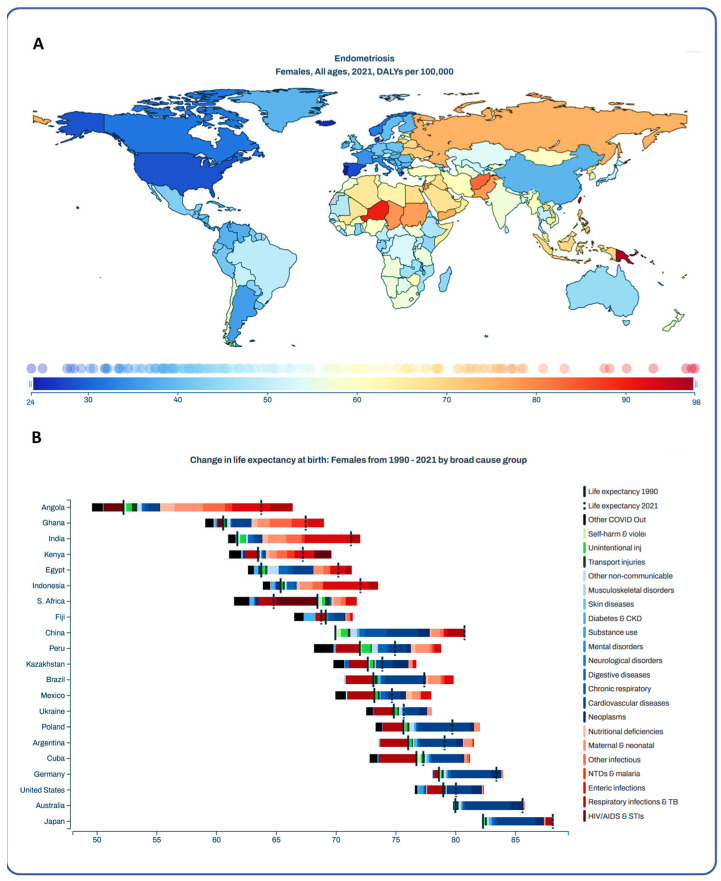
Age-standardized Disability-Adjusted Life Years (DALY) of women suffering from endometriosis and changes in life expectancy by country. Panel (**A**) shows the global DALYs for endometriosis per 100,000 women in 2021. Panel (**B**) shows the changes in life expectancy from 1990 to 2021 among women in different countries.

**Figure 6:**
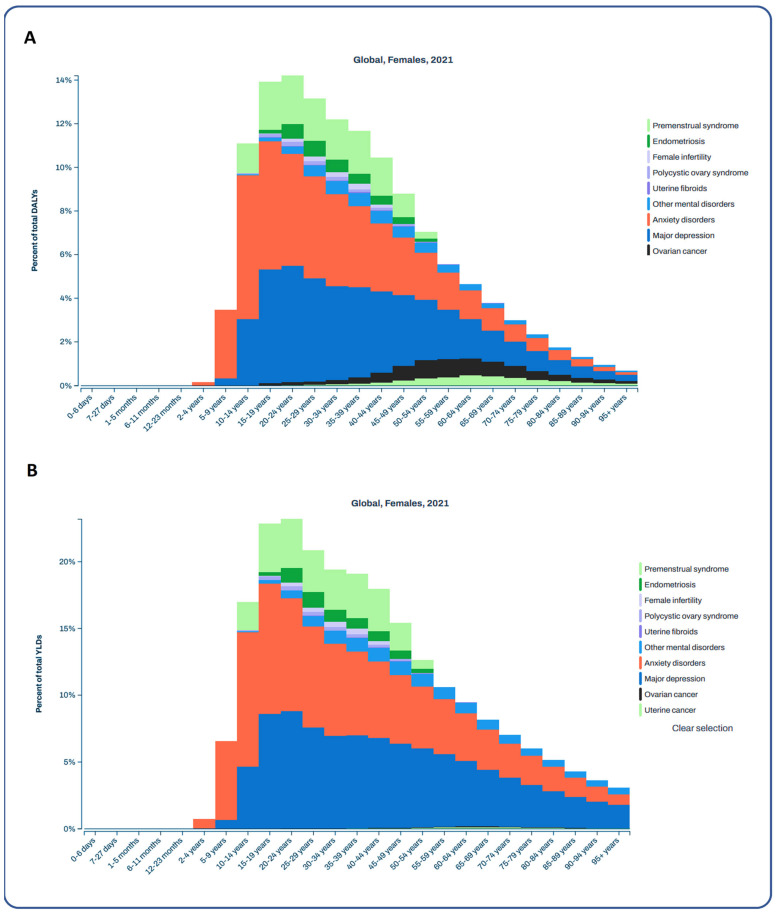
Percentages of DALYs and YLDs of women suffering from premenstrual syndrome, endometriosis, female infertility, polycystic ovary syndrome, uterine fibroids, ovarian cancer, uterine cancer, anxiety disorders, major depression, and other mental disorders. Panel (**A**) shows the percentage of total DALYs. Panel (**B**) shows the percentage of total YLDs.

According to the GBD database, the age-standardized Disability-Adjusted Life Years (DALY) for endometriosis by country differ globally (Figure 5A), with the highest age-standardized DALY rates per 100,000 cases in Niger (90.07, 95% UI: 50.09–140.04) and Papua New Guinea (96.7, 95% UI: 54.07–154.62). In contrast, the lowest age-standardized DALY rates for endometriosis were observed in Portugal (23.65, 95% UI: 13.53–36.89) and Iceland (24.89, 95% UI: 13.61–39.16). Notably, the mean life expectancy in women has changed globally over the last three decades (Figure 5B), with the lowest mean life expectancy in 2021 in South Africa and Angola, and the highest in Japan. Mental disorders, neurologic disorders, and neoplasms, including gynecologic neoplasms, are critical causes of death globally among women of all ages (Figure 5B). In 2021, endometriosis, premenstrual syndrome, anxiety, and other mental disorders represented the highest percentages of DALYs, ranging from 9.89% to 14.05%, with DALYs ranging most frequently between 10–29 years (Figure 6A). In contrast, the highest rates of total YLDs for women with endometriosis were 19.98%, for anxiety 17.21%, and for major depression 8.12%, with mean YLDs of 15–24 years (Figure 6B) in 2021. With an increase in DALYs and YLDs, the percentages of DALYs and YLDs decrease, respectively, for all aforementioned diseases.

## 3. Discussion

This investigation offers a compelling multi-faceted view into endometriosis, commencing with specific genetic alterations and extending to its profound global impact on disability and mental health. Our findings, spanning gene expression patterns and Disability-Adjusted Life Years alongside Years Lived with Disability, underscore the critical psychosomatic dimension of endometriosis, revealing a crucial interplay between biological markers, chronic pain, and significant mental health challenges like depression and anxiety, which is in line with previously published articles [6,27,28].

**Figure 7:**
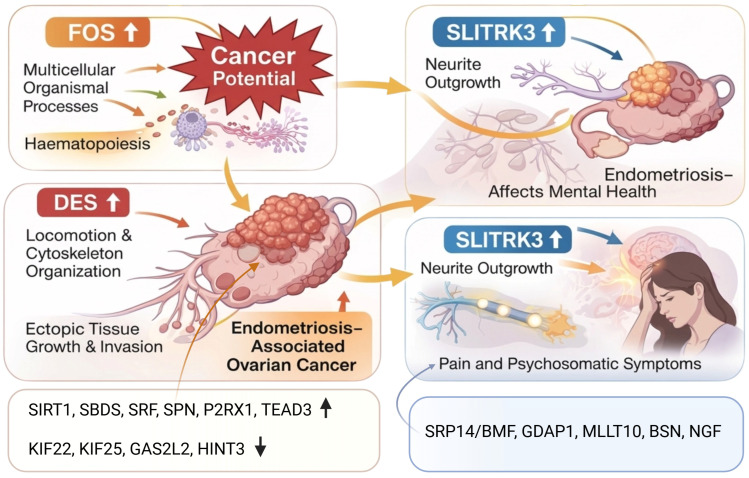
Scheme showing the potential impact of the presented differently expressed genes on the path mechanism of endometriosis, as well as endometriosis-related symptoms and sequelae. Created in Biorender. Sylvia Mechsner. (2025) https://app.biorender.com/illustrations/canvas-beta/69412c64e3fb9990f7a76a14 (access date: 16 December 2025).

The initial genetic analysis, as shown in Figure 2, Figure 3 and Figure 4, provides valuable insights into potential molecular mechanisms underlying endometriosis pathogenesis and related pain. The significant upregulation of genes such as *FOS* and *DES* in endometriosis patients, compared to controls, suggests dysregulated cellular processes critical for tissue organization, motility, and even hematopoietic functions (Figure 7). In particular, FOS, notably linked to the regulation of multicellular organismal processes and hematopoiesis, has been reported to enhance the malignant potential in endometrial stromal cells to turn into endometriosis-associated ovarian cancer [9,29], which is a critical point and concern influencing patients’ mental health, too. Its regulation by the MAPK/ERK signaling pathway, in response to oxidative stress, can also promote the proliferation of ectopic endometrial cells and inhibit apoptosis in endometriosis [30]. *FOS* is also a transcription factor that mediates the control of the endometrial cycle by sex steroids. Abnormalities in steroid hormone regulation and gene transcription, such as those seen in endometriosis, are pivotal to endometrial function [31]. As this study has shown, the gene *DES*, which encodes the protein Desmin associated with locomotion and cytoskeleton organization, was upregulated in endometriotic tissue, suggesting a biological environment conducive to the complex cellular activities observed in endometriosis, such as ectopic tissue growth and invasion (Figure 7). Furthermore, the identification of upregulated genes, such as *SIRT1*, *SBDS*, *SRF*, *SPN*, *P2RX1*, *TEAD3*, and *SLITRK3*, alongside downregulated genes, including *KIF22*, *KIF25*, *GAS2L2*, and *HINT3*, highlights a broad transcriptional reprogramming within endometriosis-affected tissues. Interestingly, the *SLITRK3* gene encodes a member of the Slitrk family of structurally related transmembrane proteins, which are involved in controlling neurite outgrowth [32]. This sheds light on the pain-related and psychosomatic symptoms of endometriosis (Figure 7).

Crucially, the clustering analysis, which reveals pain-related genes such as *SRP14/BMF*, *GDAP1*, *MLLT10*, *BSN*, and *NGF* (Figure 7), further solidifies the genetic basis for the chronic and often debilitating pain experienced by patients. These mentioned genes have also been investigated by Rahmioglu and coworkers [28], who emphasized their role in pain perception and maintenance. Notably, this genetic predisposition to pain is a pivotal link in understanding the psychosomatic experience of endometriosis, demonstrating that the biological foundation of pain significantly contributes to the patient’s subjective suffering and subsequent psychological distress. The clustering of *KRAS* and *ADAMTS19*, genes known to be involved in endometriosis-associated ovarian cancer, also provides a molecular foundation for potential malignant transformation, emphasizing the disease’s multifaceted impact [33,34].

Beyond molecular insights, the DALYs and YLDs data provide an essential and quantitative measure of the global burden of endometriosis, directly connecting the physical pathology with profound psychosomatic outcomes [35]. As shown in Figure 5, the significant age-standardized DALY rates, particularly high in regions such as Niger and Papua New Guinea, underscore the substantial impact of endometriosis on healthy life years lost worldwide. More critically for a psychosomatic perspective, the data explicitly highlight the significant contribution of mental health conditions and the need for adequate psychotherapy for endometriosis patients, which has also been reported in other studies [27,36,37]. The effective translation of this understanding into clinical practice is often hampered by significant disparities in access to healthcare and socioeconomic status. Despite affecting an estimated 190 million women globally, timely and appropriate care faces numerous barriers [22,38,39]. Notably, socioeconomic factors critically influence access to quality care. Healthcare disparities, often tied to socioeconomic status, make specialized treatments financially prohibitive for many patients [38,40]. This impacts not only diagnosis and physical treatments but also access to crucial mental health services. Research indicates that the diagnosis and treatment of endometriosis are significantly influenced by socioeconomic factors and ethnicity, resulting in differential access to care and treatment options [40,41]. Patients from lower socioeconomic backgrounds, for instance, are among those whose experiences are less investigated in research, indicating a broader systemic oversight [42]. Furthermore, regions with limited resources, such as many low- and middle-income countries, face pronounced challenges including restricted access to essential medications, surgical interventions, and fertility preservation techniques [43]. These systemic inequities exacerbate the already significant psychosocial burden of endometriosis, which is associated with impaired quality of life, anxiety, depression, and stigma [44,45,46]. Addressing these deep-seated socioeconomic and healthcare access barriers is paramount for alleviating the global burden of endometriosis and ensuring comprehensive, patient-centered care.

In 2021, endometriosis, premenstrual syndrome, anxiety, and other mental disorders collectively represented a significant proportion of DALYs (9.89% to 14.05%), frequently accounting for 10–29 years of life lived with disability. Even more strikingly, women with endometriosis experienced the highest rates of total YLDs at 19.98%, with anxiety contributing 17.21% and major depression 8.12%, equating to mean YLDs of 15–24 years. This robust evidence shows that endometriosis imposes a profound burden far beyond physical symptoms alone, as it is deeply intertwined with mental health, where anxiety and depression, acting independently and synergistically, drive a significant share of overall disability. Our findings align with those of Meyrose and coworkers [47], who evaluated treatment expectations in women with suspected endometriosis through a psychometric lens.

The chronic nature of endometriosis, characterized by persistent pain and its associated genetic underpinnings, forms a crucial foundation for the observed high rates of depression and anxiety. The constant battle with pain, coupled with diagnostic delays, impact on fertility, and limitations on daily activities, can profoundly erode a patient’s mental well-being. This is where the psychosomatic perspective becomes paramount: the physical manifestation of endometriosis, rooted in genetic expression and cellular dysregulation, directly contributes to psychological distress, which in turn can modulate pain perception and overall quality of life. The observed inverse relationship between increasing DALYs/YLDs and their respective percentages for all aforementioned diseases may suggest that as the overall burden of disability from these conditions increases, other severe conditions begin to contribute more, or it could reflect the long-term, compounding effect of chronic illness on an individual’s life trajectory.

Our findings indicate a strong correlation between the severity of endometriosis symptoms and the prevalence of psychological comorbidities, particularly chronic anxiety and clinical depression, among affected individuals. This suggests that the chronic pain and systemic inflammation associated with endometriosis significantly contribute to the development and exacerbation of mental health issues, forming a complex bidirectional relationship where physiological distress amplifies psychological burden and vice versa [48].

Furthermore, the pervasive stigma associated with endometriosis, often leading to dismissal of symptoms and delayed diagnosis, intensifies this psychological distress, influencing mental health outcomes and quality of life [4,49]. This persistent psychological pressure can manifest as heightened pain perception, increased fatigue, and diminished coping mechanisms, further perpetuating the cycle of suffering [49]. The social and medical dismissal of symptoms, coupled with the invisible nature of the disease, frequently leads to feelings of isolation and invalidation, compounding the psychological impact on patients [11]. This chronic psychological burden, characterized by elevated stress and anxiety, is hypothesized to contribute to increased symptom severity and disease progression through neuroendocrine–immune pathways.

## 4. Materials and Methods

### 4.1. Turku Endometriosis Database

To investigate the expression patterns of specific genes related, among others, to pain and malignant transformation within clinical endometriosis samples and their healthy counterparts, we utilized the raw data from the EndometDB Turku Database [24]. This sophisticated, interactive web-based platform provides access to mRNA expression data from 115 patients with endometriosis and 53 control individuals, encompassing over 24,000 genes and their associated clinical features. Diagnosis of endometriosis was definitively established through laparoscopy or laparotomy, with histopathological examination of biopsies serving as the confirmation. To establish a baseline, laparoscopy was performed to rule out endometriosis in healthy women undergoing tubal sterilization. The stage of the menstrual cycle at the time of sampling was meticulously documented using a questionnaire, endometrial histology, and serum progesterone levels. For detailed transcriptional analysis, three distinct categories of endometriosis samples were procured: Firstly, deep infiltrating endometriosis lesions, including deep rectovaginal, sacro-uterine ligament lesion, intestinal endometriotic lesions, and deep endometriotic lesions in the bladder; secondly, peritoneal endometriosis lesions, including red peritoneal endometriotic lesion, black peritoneal endometriotic lesion and white peritoneal endometriotic lesion; and lastly, ovarian endometrioma samples were taken into consideration. Moreover, endometrium samples were collected from both patients and healthy controls, as well as peritoneum samples from both healthy controls and patients. EndometDB enabled a precise comparative analysis of mRNA expression levels between control endometrium and various endometriosis-affected tissues, including ovarian endometrioma, peritoneal lesions, and deep endometriosis. Comprehensive details regarding the raw data and the database itself are thoroughly documented in the original publication [24].

### 4.2. Global Burden of Disease Database

To provide robust insights, this study leveraged the extensive Global Burden of Disease (GBD) 2021 database [25]. This invaluable resource offers comprehensive estimates encompassing incidence, prevalence, mortality, risk factors, years lived with disability, years of life lost, and Disability-Adjusted Life Years across 369 diseases and injuries. The data spans both genders, 23 age groups, 21 regions, and 204 countries and territories, with a historical scope extending from 1990 to 2021. Ensuring internal consistency across various dimensions, these estimates are meticulously generated using Dis-Mod-MR 2.1, a sophisticated Bayesian meta-regression modelling tool (software version number: 1 January 2024). For a comprehensive understanding of the methodology, please refer to the detailed work by the GBD 2021 Disease and Injury Incidence and Prevalence Collaborators [26].

### 4.3. Disease Burden

This study assessed the pervasive burden of endometriosis and its related conditions, using Disability-Adjusted Life Years as the primary metric. DALYs, a composite measure that integrates Years of Life Lost and Years Lived with Disability, offer an unparalleled scope for quantifying the total health deficit incurred by endometriosis. This standardized approach ensures robust comparisons of the disease’s global and temporal health impact, highlighting the urgent need for effective interventions.

### 4.4. Statistical Analysis

The differential gene expression data (EndometDB) were calculated using linear modeling in R (software version number: 4.3.3). Briefly, log2-transformed gene expression counts were analyzed using linear modeling with the function expression ~ condition to generate log2 fold changes and *p*-values for each gene. Significant genes were determined using a *p*adj cutoff of 0.1. Enrichment of substantial genes was performed using the clusterProfiler package in R (software version number: 4.10), specifically the enrichGO function, with the list of substantial genes as the gene list and the list of all quantified genes as the universe. Enrichment plots were generated using the GOplot package in R (software version number: 1.0.2), with the top 10 hits from GO term enrichment used for the enrichment plot. *p*-values were then adjusted using false discovery rate correction, * *p* < 0.05, ** *p* < 0.01, *** *p* < 0.001, and **** *p* < 0.0001.

## 5. Conclusions

In conclusion, our findings underscore the importance of adopting a holistic, psychosomatic approach to managing endometriosis. This piece shows clearly that a patient’s psychosomatic suffering due to endometriosis should be taken seriously. The identified genetic markers related to pain provide a biological basis for the profound physical suffering, while the robust DALYs and YLDs data clearly quantify the devastating impact on mental health, particularly highlighting the significant burden of depression and anxiety among affected individuals. This comprehensive understanding is crucial for developing integrated psychosomatic therapeutic strategies that not only target the biological mechanisms of the disease but also proactively address the pervasive psychological distress, ultimately aiming to improve the overall quality of life for women living with endometriosis. A collaborative and patient-centric therapeutic approach helps patients feel empowered. This being said, there is an ultimate need for early integration of psychological support to help patients manage the emotional burden associated with their diagnosis and treatment of endometriosis. One of the significant issues patients have to deal with is that many of them suffer from symptoms that are obviously endometriosis-related. Still, many physicians have dismissed these as signs of endometriosis. After years of suffering, they finally receive an endometriosis diagnosis. What is even more absurd is not only getting a diagnosis of endometriosis after years of waiting, but also getting a diagnosis of endometriosis-associated cancer.

Future research could focus on cellular and molecular underpinnings of endometriosis-associated pain and its intersection with psychological distress, such as genetic marker validation and functional studies or neuroendocrine–immune interaction models. Moreover, animal models could be interesting for investigating the systemic effects of endometriosis, particularly the complex interplay between physical pain and mental health. Upon comprehensive pain and psychological assessments, existing rodent models could be refined to better understand both the physical pain and psychological manifestations of endometriosis.

## Data Availability

The data presented in this study are openly available in the EndometDB Turku Endometriosis Database and the Global Burden of Disease Database, both of which are available at: https://endometdb.utu.fi/ (access date 20 July 2025) and https://ghdx.healthdata.org/gbd-2021 (access date 20 July 2025).

## Abbreviations

The following abbreviations are used in this manuscript:

DALYs	Disability-Adjusted Life Years
DEGs	Differentially Expressed Genes
EAOC	Endometriosis-Associated Ovarian Cancer
GBD	Global Burden of Disease Study
GO	Gene Ontology
YLDs	Years Lived with Disability
